# Hydroxyurea Treatment in Transfusion-Dependent β-Thalassemia Patients

**DOI:** 10.5812/ircmj.18028

**Published:** 2014-06-05

**Authors:** Mohammad Reza Bordbar, Samir Silavizadeh, Sezaneh Haghpanah, Roza Kamfiroozi, Marzieh Bardestani, Mehran Karimi

**Affiliations:** 1 Hematology Research Center, Shiraz University of Medical Sciences, Shiraz, IR Iran; 2 Department of Library and Information Science, Khuzestan Science and Research Branch, Islamic Azad University, Ahvaz, IR Iran

**Keywords:** Hydroxyurea, Blood Transfusion, β-Thalassemia

## Abstract

**Background:**

β-Thalassemia is an inherited hemoglobin disorder caused by defective synthesis of ß-globin chains. Hemoglobin (Hb) F induction is a possible therapeutic approach which can partially compensate for α and non-α globin chains imbalance.

**Objectives:**

We aimed to investigate the efficacy and safety of Hydroxyurea (HU) in diminishing transfusion requirements of patients with β-thalassemia major in Southern Iran.

**Patients and Methods:**

In this single-arm clinical trial, all transfusion-dependent β-thalassemia patients older than two years old (n = 97) who had inclusion criteria of the study and had been registered for at least six months in Dastgheib thalassemia outpatient clinic (a referral center affiliated to Shiraz University of Medical Sciences) were evaluated from October 2010 to December 2011. The patients were treated with HU with a mean dose of 10.5 mg/kg for a mean duration of 8 months (range 3-14 months). Transfusion needs and Hb levels were compared before and after HU treatment.

**Results:**

The mean volume of blood transfusion decreased significantly following HU treatment (0.71 mL/kg/day vs. 0.43 mL/kg/day, P < 0.001). Two-thirds of the patients showed good and partial response. No serious adverse reaction was observed except persistent neutropenia in two patients.

**Conclusions:**

Hydroxyurea can be safely used in some transfusion-dependent β-thalassemia patients to decrease their transfusion needs.

## 1. Background

β-Thalassemia, as the most prevalent hereditary blood disorder, results from defective synthesis of β-globin chain which leads to ineffective erythropoiesis due to imbalance of α and non-α globin chains production (1). Patients with β-thalassemia major need regular blood transfusion, as well as iron chelating agents in order to keep up a normal life.

Reactivation or augmentation of fetal hemoglobin (HbF), is a possible therapeutic approach for patients with β-thalassemia, as it can compensate for defective β-globin chain production (2). Hydroxyurea (HU), which is a ribonucleotide reductase inhibitor, is capable of increasing HbF production and partially correcting α and non-α globin chains imbalance, thus ameliorating the hemolytic symptoms of these patients (3).

Although HU was initially approved for reduction of painful crises in sickle cell patients (4-9), its efficacy in induction of transfusion independence in patients with β-thalassemia intermedia was also studied by several investigators (10-16). However, the experiences regarding the efficacy of HU in patients with β-thalassemia major are limited with controversial responses (17-25).

## 2. Objectives

The aim of this study was to evaluate the clinical response of transfusion-dependent β-thalassemia patients to HU in southern part of Iran where β-thalassemia is most prevalent.

## 3. Patients and Methods

This single-arm clinical trial study was conducted at Hematology Research Center in Shiraz, Southern Iran from October 2010 to December 2011. We included all transfusion-dependent β-thalassemia patients who were older than two years old and had been registered for at least six months in Dastgheib thalassemia outpatient clinic, which is a referral center affiliated to Shiraz University of Medical Sciences.

They were collectively labeled as β-thalassemia major as their measured Hb values were less than 7 g/dL in two consecutive times (1-2 months apart) within the first two years of their lives. Since then, they were put on regular transfusion every 2-4 weeks to keep their pretransfusion Hb above 9.5 g/dL.

 The diagnosis of β-thalassemia was based on complete blood count and hemoglobin electrophoresis. Informed written consent was taken from those taking part in the study. The ethics committee of the Shiraz University of Medical Sciences approved the project (code number = 2401, date: August 2010). The ethical considerations were related to the safety issues and toxicity monitoring.

Those with impaired renal function (serum creatinine > 2 times upper normal limit for age), active hepatitis B or C infection on Interferon therapy, elevated liver enzymes more than 2 times normal, thrombocytopenia lower than 100,000/µL, neutropenia under 1500/µL, and those who took HU less than three months were excluded.

Out of 554 registered patients in our center, 156 patients were accepted to participate in the study. By implementing the inclusion and exclusion criteria, 121 patients were eligible to be started with. However, 24 patients, who consumed the drug for less than three months were excluded from the study. Finally, 97 cases with mean age of 20.27 ± 8.3 years (range 2-50 years) (42 males, 55 females) remained in this study.

Hydroxyurea (Hydrea, Bristol-Myers Squibb, Italy) was started at a mean dose of 10.58 ± 1.57 mg/kg/day (range 8-14 mg/kg/day). The patients were visited every two weeks by a fixed attending hematologist for evaluation of their clinical and laboratory response. They were examined clinically for any sign of new onset of extramedullary hematopoiesis, including hepatosplenomegaly or facial bones changes. Complete blood count (CBC) was checked by a Sysmex KX-21 analyzer (TOA system, Japan) in every visit. Liver and renal function tests were performed monthly using Merck kits (Germany). Serum ferritin levels were measured by enzyme-linked immunosorbent assay (Dynex; Monobind Inc. Lake Forest, CA) every three months. Hemoglobin F was checked with alkaline denaturation method before starting HU and at the completion of the study.

All equipment used for the measurement of clinical variables was calibrated. At every follow-up, the remaining capsules of HU were counted to evaluate therapeutic compliance. Toxicity monitoring was done according to the descriptions in the exclusion criteria. In case of any sign of drug toxicity, HU was withheld temporarily and restarted when the tests normalized.

Our primary endpoint was transfusion requirements during the 14-month periods on HU compared to the same periods before entering the study. Therefore, every patient served as historical control for him/herself. Our policy for packed-cell transfusion was Hb value less than 7 g/dL, and/or signs and symptoms of anemia such as lethargy, loss of energy, loss of appetite, and low back pain, or coexisting infection.

Response criteria were defined as:

1. Excellent responders, if there were no need for further transfusion,
2. Good responders, if the transfusion requirements reduced more than 50% compared to the control period,
3. Partial responders, if the transfusion needs decreased 25-50%, and
4. No responders, if the transfusion needs reduced less than 25%. The first two groups were then collectively classified as “responders group”.

### 3.1. Statistical Analysis

Data analysis was done by SPSS v. 17 (SPSS Inc. Chicago, IL, USA). Normality of data was assessed by Kolmogorov-Smirnov test. To compare quantitative variables in patients before and after treatment with HU, paired samples t-test and Wilcoxon signed ranks test were used. Comparison of quantitative variables among three groups was done by 1-way analysis of variance (ANOVA) and Kruskal-Wallis Test. Chi-square test was done to compare ratios among different groups. Two-sided P value < 0.05 was considered statistically significant.

### 3.2. Sample Size Calculation

Based on the estimated decrease in transfusion need of about 0.2 mL/kg/day after HU consumption, standard deviation = 0.3, α = 0.05, and power at 95%, sample size was calculated as 60 patients. However, we decided to evaluate all patients with inclusion criteria during the study period (n = 121). Finally, 97 patients were evaluated after drop out.

## 4. Results

The mean follow-up period of patients on HU was 7.72 ± 3.59 (range 3-14) months. Fifty-four patients (56.3%) were splenectomized. Thirty-four patients (35.1%) continued to take the drug till the end of the study, and the remaining 63 patients discontinued using HU before 14 months.

 The reasons for drug withdrawal in decreasing order were: 1) recurrent drops in hemoglobin levels below 7 g/dL necessitating packed-cell transfusion (63.5%), 2) fear of facial skeletal changes and self-inclination (19%), 3) severe persistent lethargy and tiredness (8%), and other causes (9.5%).

The most common side effects observed were temporary rise of liver enzymes above 2 times normal (16.5%), and transient neutropenia (14.5%), which resolved after a short course of drug withdrawal. Two patients experienced persistent neutropenia which led to their exclusion from the study.

Table 1 show the mean pretransfusion Hb, serum ferritin, HbF, and transfusion needs before and after HU therapy. Although the mean pretransfusion Hb level after HU therapy was lower compared to pre-HU trial (P < 0.001), the transfusion needs decreased significantly after drug consumption (P < 0.001). The mean serum ferritin level decreased significantly in the post-HU periods (P = 0.021). Hemoglobin F level increased after starting the trial, which was statistically significant (P < 0.001). The need for packed-cell transfusions was not influenced by age, splenectomy, serum ferritin, nucleated red blood cells (NRBCs) in the peripheral blood smear, and duration of HU consumption (P > 0.05).

**Table 1.** Hematological Variables Before and After Hydroxyurea Treatment ^a,b,c^

Variables	Pre-HU Treatment	Post-HU Treatment	P Value
**Pre-transfusion Hb, g/dL**	9.53 ± 1.1	8.28 ± 0.73	< 0.001
**Transfusion, mL/kg/d**	0.71 ± 0.21	0.43 ± 0.21	< 0.001
**Transfusion interval, d**	14 ± 3	28 ± 7	0.012
**Median**	15	16.2	
**Serum Ferritin, ng/mL**	2498.25 ± 1980.77	2214.72 ± 1950.44	0.021
**Median**	1864	1463	
**Hb F, g/dL**	1.1 ± 0.3	5 ± 1.1	< 0.001

^a^ Abbreviations: HbF, hemoglobin F; HU, hydroxyurea.

^b^ Data are presented as mean ± SD.

^c^ Serum ferritin levels and transfusion interval did not show normal distribution at the studied population; thus, nonparametric test was done, and median value was also reported for both.

Out of 97 thalassemia patients who entered the study, 95 were suitable for response assessment because two patients had irregular follow-up and missed related data. According to the response criteria defined earlier, 31 patients (32.6%) were regarded as responders. Six thalassemia patients (6.3%) became transfusion independent. Thirty-three patients (34.8%) showed partial response, and the rest of them (32.6%) were classified as non-responders. None of the measured variables, including sex, splenectomy, before and after HU Hb, and serum ferritin levels were associated with the therapeutic response, except HbF level, which was significantly higher in the responders group (P < 0.05). The percentage of NRBCs in the peripheral blood smear tended to be higher in the responders group compared to the non-responders (57.1% vs. 32.3%), and the difference inclined to be statistically significant (P = 0.058). Patients were also divided into three groups based on HU dosage (8-10 mg/kg/day, 10.1-12 mg/kg/day, and 12.1-14 mg/kg/day). There was not a statistical significant difference among these three groups of patients with regard to the response to treatment (P = 0.279). The result of genotypic investigation was available in about half of our patients. The most frequent genotype observed in more than 90% of them was homozygous IVS II-1, though our previous report showed that response to HU is not related to β-globin gene mutation (26). As a result, we decided not to include this variable in response assessment.

## 5. Discussion

Hemoglobin F induction has been a longstanding therapeutic goal for the treatment of β-thalassemia. Three classes of HbF inducing agents have been introduced. They are hypomethylating agents (such as HU, decitabine and 5-azacytidine), histone deacetylase inhibitors (like sodium phenylbutyrate, and isobutyrate), and finally recombinant erythropoietin (2). These agents have been shown to increase total Hb levels by 1-5 g/dL above baseline if administered for at least 3-6 months (3). Hydroxyurea is the most widely accepted HbF inducer, and its efficacy was investigated in several studies.

Ansari et al. (18) showed an 80% response rate in 152 transfusion-dependent thalassemia patients taking HU. The therapeutic response was obvious after a mean of 65 days of the drug consumption. Alebouyeh (17) found similar response, and his patients exhibited a rise in post-HU treatment Hb, as well as a decrease in serum ferritin. Another study conducted by Italia (22) showed a very good responsiveness in thalassemia intermedia patients, (74% good response). However, only a third of his patients with thalassemia major had more than 50% reduction in their transfusion requirements. Similarly, Bradai (20) tried HU in 45 patients with thalassemia major with a mean dose of 17 mg/kg. About half of his patients exhibited more than 70% reduction in their transfusion requirements which by our definition meant good response. He concluded that the higher age at first transfusion, higher baseline Hb, and splenectomy were correlated with a better response. However, increasing the dose of HU had no impact on the transfusion needs. Another study conducted by Yavarian (15) proved the effectiveness of HU in 133 transfusion-dependent thalassemia patients, as it resulted in elimination of transfusion in more than 60% of cases and occasional transfusion (i.e. 1-2 per year) in another 23% of patients. XmnI polymorphism had the most outstanding role in clinical responsiveness of his patients. In our study, the patients could be equally divided into three groups (Table 2). A third showed a good response to HU and their transfusions either stopped (in six cases) or reduced more than 50%. The second group composed of those whose transfusion requirements diminished less than 50%, but they were transfused more infrequently and with longer intervals. The rest of the patients either did not respond or showed a poor response. In other words, about two-thirds of our patients were more or less responsive to HU, which is similar to most previous studies (15, 17, 18, 20, 22, 23, 25, 26). Although the definition of response varies in different studies, we showed (like many other investigators) that HU may bring about a state of transfusion independence in those known as responders. This finding is similar to the result of most other studies mentioned earlier except the fact that it was not accompanied by any rise of Hb, even in the good responders. Since the Hb threshold for packed cell transfusion differed before and after HU treatment, the mean pretransfusion Hb could not be compared between two groups. Likewise, there is a similar debate regarding the transfusion requirements between two groups. As it was stated in the method section, all of our patients experienced Hb levels less than 7 g/dL before starting regular transfusion when they were under two years old. Accordingly, if our patients did not show any clinical response to HU, we expected to notice more Hb drops to below 7 g/dL after cessation of regular transfusion, while their Hb values remained above 7 g/dL despite not being transfused. As a result, the responders needed less transfusion compared to pre-HU treatment, even though they were more anemic.

**Table 2.** Comparison of Demographic, Clinical and Laboratory Data Among Different Groups of Transfusion-Dependent β-Thalassemia Patients ^a,b,c^

Variables	Good Responders, n = 31	Partial Responders, n = 33	Non Responders, n = 31	P Value
**Sex, male, No. (%)**	17 (54.8)	15 (45.5)	9 (29)	0.116
**Splenectomy, No. (%)**	17 (54.8)	11 (33.3)	14 (45.2)	0.222
**Pre-HU Hb, g/dL**	9.77 ± 0.95	9.61 ± 1.04	9.19 ± 1.23	0.114
**Post-HU Hb, g/dL**	8.55 ± 0.76	7.96 ± 1.57	8.06 ± 0.70	0.079
**Pre-HU Ferritin, ng/mL**	2305 ± 1657	2987 ± 2288	2319 ± 2025	
**Median** ^**d**^	2008	2176	1446	0.547
**Post-HU Ferritin, ng/mL**	2039 ± 1479	2635 ± 2299	1818 ± 1474	0.777
**Median** ^**d**^	1792	1816	1324	
**HU dose, mg/kg/d**	10.62 ± 1.68	10.96 ± 1.45	10.21 ± 1.56	0.169
**HU duration, w**	9 ± 3.82	7.24 ± 3.47	7.10 ± 3.32	0.066
**NRBC, yes, No. (%)**	13 (41.9)	5 (15.2)	10 (32.3)	0.058
**Pre-HbF, g/dL**	1.1 ± 0.4	1.2 ± 0.3	1.2 ± 0.2	0.225
**Post-HbF, g/dL**	7.4 ± 1.2	4.1 ± 1.3	3.5 ± 0.9	< 0.05^ c^

^a^ Data are presented as mean ± SD.

^b^ Abbreviation: Hb, hemoglobin; HU, Hydroxyurea, NRBC, nucleated red blood cells.

^c^ Post-HU HbF level was significantly higher in good responders compared with partial and non-responders (P < 0.001). Also in partial responders, a significant higher level of post-HU HbF was observed compared with non-responders (P = 0.036).

^d^ Because before and after serum ferritin levels did not have normal distribution at the studied population, nonparametric test was done and median value was also reported for both.

Among the measured variables, only HbF had a direct impact on the therapeutic response (Table 2). It seems that a subset of patients who respond better to HbF induction can take the most benefit from HU treatment. Nevertheless, some researchers proved that the increase in HbF did not always correlate with a hematologic response which in turn supports this hypothesis that HU may have a more general role in ß-thalassemia patients besides HbF induction (27). The amount of NRBCs in the peripheral blood smears tended to be higher in the responders group (57.1% vs. 32.3%, P = 0.05). As we did not detect any sign of extramedullary hematopoiesis such as progressive hepatosplenomegaly or skeletal bone deformities (rather than those which were already established), it verifies that HU could keep transfusion independence by implementing more effective bone marrow erythropoiesis.

Although the amount of NRBCs in the peripheral blood is an indirect sign of ineffective erythropoiesis, it seems that HU induces maturation of NRBCs up to later stages of normoblasts and better RBC survival, thus reducing ineffective erythropoiesis. There is a large body of evidence that HU may improve erythrocyte morphology and deformability in sickle cell disease leading to reduced hemolysis, though similar studies in patients with β-thalassemia are limited (27, 28). As the patients taking HU had a more sense of well-being compared to the time they were regularly transfused, we decided to transfuse them less frequently and with Hb values, less than 7 g/dL, unless they were symptomatic. It may account for fewer transfusion needs despite the lower Hb values in post-HU treatment, and would justify the paradox noted between the post-HU Hb levels and the transfusion requirements after starting HU.

We administered HU with a mean dose of about 10 mg/kg/day, similar to Karimi’s, Yavarian’s and Zamani’s studies (15, 25, 26), but much lower than some other investigators (17-20, 22, 29). We concluded that although increasing the dose of HU was positively correlated with fewer transfusion needs (r = 0.2, P = 0.03), it had no impact on the therapeutic response. Therefore, it may be logical to start the drug with the dose of 10 mg/kg/day to avoid possible myelosuppression sometimes observed with higher doses of HU, and increase the dosage if there is a poor clinical response. The duration of treatment does not affect the response rate, so if HU therapy does not lead to a satisfactory response after treatment with optimal doses for about 3-6 months, it should be determined whether or not its usage would be beneficial. While there is an emerging concern about the long-term efficacy of HU therapy in β-thalassemia patients beyond 12 months, as it was proposed that HU may adversely affect the ability of hematopoietic stem cells to undergo erythroid differentiation (27), our experience with HU in a large number of patients with β-thalassemia intermedia proved the long-term efficacy and safety of this drug (13, 14, 30).

Although this is one of the few prospective studies investigating the role of HU in inducing transfusion independence in β-thalassemia major patients, our study faced some limitations. We could not define the specific mutations of the β-globin genes or XmnI polymorphisms in all of our patients due to financial problems. Although most of the previous studies showed some correlations between XmnI polymorphisms or some specific genetic mutations and therapeutic response (15, 16, 20, 22, 23), others failed to demonstrate any significant relationship (10, 18, 26). It seems that the interaction of many factors, including different genetic mutations, α and γ globin chains production, XmnI polymorphism and other biochemical factors contribute to the therapeutic response to HU.

The other limitation of our study was the relatively short study period to assess the long-term safety and efficacy of the drug. As most of our patients refused to continue the trial due to the fear of bone deformity or the other possible drug adverse effects (though observed infrequently), we had to stop the trial after about 14 months and analyze the short-term effects of the drug. An education program is needed to convince transfusion-dependent thalassemia patients to consume HU regularly and for a long time, since most of the thalassemia major patients are adapted to transfusion, and they hardly accept to share in other treatment regimens. It is noteworthy that we continued the study with a smaller group of patients with better compliance to evaluate the long-term efficacy and safety of the drug, and the results will be issued in the future. In conclusion, HU as the most widely studied HbF inducer can be safely prescribed to some of transfusion-dependent β-thalassemia patients in order to diminish their transfusion requirements and bring about a feeling of well-being.
